# Rheumatic heart disease in Africa: is there a role for genetic studies?

**DOI:** 10.5830/CVJA-2015-037

**Published:** 2015

**Authors:** Ana Olga Mocumbi

**Affiliations:** Instituto Nacional de Saúde and Universidade Edurado Mondlane, Maputo, Moçambique

**Keywords:** rheumatic fever, rheumatic heart disease, genetic susceptibility

## Abstract

Rheumatic heart disease (RHD) constitutes a leading cause of premature death and incapacity in Africa, where it is encountered in younger people, and shows a much faster and more malignant course than that seen in Europe or North America. While it is well established that RHD is a consequence of recurrent, untreated group A β-haemolytic streptococcal infections (GAS), the pathogenesis is incompletely understood, and the variation in natural history and phenotypes are not fully explained. In Africa patients are rarely diagnosed with acute rheumatic fever (ARF). They usually present in the late stages of RHD, with the severe and virulent forms occurring at early ages, therefore leading to high morbidity and mortality in young patients.

## Epidemiology of ARF and RHD

The global burden of disease caused by rheumatic fever (RF) and rheumatic heart disease (RHD) currently falls disproportionately on children living in the developing world and in marginalised communities where poverty is widespread. Acute rheumatic fever (ARF) follows in 0.4 to 3.0% of cases of group A β-haemolytic streptococcal pharyngitis (GAS) in children. It is thought that as many as 39% of patients with acute rheumatic fever may develop varying degrees of pancarditis with associated valve regurgitation and heart failure and in some cases death. RHD is the only long-term consequence of ARF, and the most serious.^1^ Progression to chronic RHD is determined by several factors, among which, repeated episodes of rheumatic fever seem to be the most important.

The World Health Organisation (WHO) reported trends in the incidence and prevalence of ARF and RHD for each continent,^2^ based on literature from 100 countries around the world between 1970 and 2009. These studies on ARF incidence and RHD prevalence used population-based screening, national health registries, prospective disease surveillance, surgical series, autopsy series, and retrospective reviews of hospital admissions or discharges for ARF or RHD. However, data from Africa are scarce and do not capture the entire timeframe. According to this study, the reported incidence of ARF is decreasing in all WHO regions, except for the Americas and the western Pacific, where it appears to be increasing. The prevalence of RHD is increasing in all regions except for Europe, where it appears to be decreasing.^2^

A systematic review and meta-analysis of population-based studies published between January 1993 and June 2014 recently reported on the prevalence of RHD among children and adolescents assessed in 37 populations, six of which were from Africa.^3^ It revealed a prevalence of RHD detected by cardiac auscultation at 2.9 per 1 000 individuals and by echocardiography at 12.9 per 1 000 people, with substantial heterogeneity between individual reports for both screening modalities. Prevalence of clinically silent RHD in this study (21.1 per 1 000) was about seven to eight times higher than that of manifest disease (2.7 per 1 000). Prevalence progressively increased with advancing age, from 4.7 per 1 000 at age five years to 21.0 per 1 000 people at 16 years. There was no gender-related differences in prevalence; an association was found between social inequality expressed by the Gini coefficient and prevalence of RHD (*p* = 0.0002).^3^

The exact incidence and prevalence of RHD in Africa are unclear because of the recognised differences in epidemiology between countries, availability of diagnostic approaches, differences between rural and urban environment, age groups included in the study, and more importantly, lack of knowledge about the outcome of the mild lesions found in community-based studies on asymptomatic children. In Western countries, marked reduction in RHD prevalence occurred with improvement in health systems (education of health professionals for quicker diagnosis and correct management with antibiotics) and socio-economic status (less overcrowding, education of the population).

RHD was the leading cause of death 100 years ago in people aged five to 20 years in the United States but, as in other developed countries, its incidence has declined.^4^ This reduction is related to the adequate treatment of streptococcal pharyngitis with penicillin, as well as less overcrowding, better sanitation and improvement in general living conditions. The incidence of ARF has dropped dramatically since the 1960s; a few localised outbreaks of GAS occurred in civilian and military populations in the 1980s.4 The reported increase in RHD prevalence2 is likely to be related to increased survival due to advances in diagnosis, and medical and surgical treatments for RHD.

RHD remains the most common cardiovascular disease in people under 25 years and is the leading cause of valve disease in developing countries.^5^,^6^ The African continent has the highest prevalence in the world,^2^ and RHD represents the most common form of acquired cardiovascular disease in children and adolescents.^7^ RHD affects between 15.6 and 19.6 million people worldwide and causes 233 000 to 492 000 deaths annually,^8^ imposing a substantial burden on the families, health systems and communities in many low-income settings.

Screening with portable echocardiography has uncovered a large burden of latent RHD among asymptomatic children in endemic regions of Africa,^9^,^10^ the significance of which remains unclear.^11^ In marked contrast, there are almost no data on ARF, probably related to low access to healthcare, inadequate resources for diagnosis of throat and skin streptococcal infection, lack of awareness of the importance of correct treatment of bacterial pharyngitis, and overall, to the absence of national prevention and control programmes. These usually allow notification of the disease and the institution of long-term secondary prophylaxis to those at risk of developing RHD.

The reduction in the burden of ARF and RHD among the less than 20% of the world’s population living in high-income countries has led to a decrease in research on rheumatic fever (RF) and RHD.^12^ Despite being a major cause of premature death and disability, the pathogenesis is still incompletely understood, the natural history is not fully explained, phenotypes have been only partially described, and some aspects of management remain debatable.

## Pathogenesis of ARF and RHD

Throat infection by GAS is the common trigger for RF/RHD. In resource-limited tropical settings however, where both impetigo and rheumatic disease are endemic, there is a growing body of opinion implicating impetigo in the pathogenesis of rheumatic fever and rheumatic heart disease.^13^ Repeated GAS infection is necessary for the first episode of ARF to occur, and similarly, RHD usually develops due to cumulative damage to the heart valves secondary to recurrent episodes of ARF.^14^

Molecular mimicry explains the triggering of RF, but an intense and sustained inflammation is needed to cause sequelae.^15^,^16^ Antigens in the cell wall and cell membrane of GAS are immunologically similar to molecules in human myosin, tropomyosin, actin, laminin and other common proteins. GAS carbohydrate epitope (N-acetyl glucosamine) and the α-helical coiled-coil streptococcal M protein structurally mimic cardiac myosin.^17^ When GAS antigens reach the blood, they are recognised by B cells in the spleen; they may also enter the lymph and be recognised by B cells in local lymph nodes.

B cells specific for GAS antigens become activated and begin to proliferate and secrete antibodies, activate complement and promote the opsonisation and phagocytosis of the bacteria.^16^,^18^ An autoimmune response is triggered in susceptible children, in whom antibodies against streptococcal antigens (mainly the M protein) cross-react with heart tissue proteins such as cardiac myosin (in the myocardium) and laminin (on valve endothelium and basement membrane).^17^ At the same time, antigens taken up at the site of infection by antigen-presenting cells become activated and migrate to local lymph nodes where they present the antigens to T cells. Activated T cells begin to proliferate and additionally stimulate B cells to produce antibodies against the GAS antigens. It is believed that both T cell and antibody crossreactions occur between GAS and host proteins.^19^

In rheumatic carditis, attachment of anti-GAS antibodies to the myocardium and valve endothelium leads to the release of inflammatory cytokines that up-regulate vascular cell adhesion molecule-1 (VCAM-1) on the valve surface endothelium; this up-regulation of VCAM-1 promotes lymphocyte adhesion to the endothelium and subsequent infiltration of lymphocytes into the valve. Both inflammatory (transforming necrosis factoralpha: TNF-α, and interferon-gama: IFN-γ) and regulatory (interleukin: IL-4) cytokines are produced, increasing local inflammatory reactions in both the myocardium and the valves. Granulomatous lesions containing lymphocytes and macrophages are formed, the so-called Aschoff nodules, which are identifiable and regarded as pathognomonic for rheumatic carditis.^20^

The initial attack with ARF increases vulnerability to reactivation of the disease, with subsequent pharyngeal infection.^21^ Exposure of the valve surface to inflammation ensures further binding of cross-reactive antibodies to the valve, leading to endocarditis, which is on the basis of rheumatic heart valve disease (RHVD),^16^ and the lack of production of regulatory cytokines may contribute to permanent valve damage.^18^. Chronic RHVD can result from a single episode of ARF, but usually follows repeated episodes of ARF, with cumulative valve damage occurring due to fibrotic healing of acute inflammatory lesions and turbulent flow induced by ongoing valve damage.^22^ The major morphological changes of the valves include commissural fusion, shortening and fusion of the chordae tendinae, and leaflet thickening.^23^

## Gaps in knowledge and management

Although the diagnosis of GAS pharyngitis may be suspected on clinical examination, several procedures are involved in its confirmation, because clinical presentation performance as a diagnostic test is low. Laboratory test availability is important, especially culture, virulence test, antibiotic sensitivity, C-reactive protein and erythrosedimentation rate.^24^ Because these examinations are expensive and time consuming, rapid antigen testing is a more attractive solution for Africa.^25^ Therefore the diagnosis of ARF relies on a high index of suspicion from health workers, a high level of awareness of the community, and laboratory criteria of recent infection or previous ARF.

Some symptoms and signs included on the Jones criteria are unspecific and may be present in various febrile conditions affecting children in Africa. Moreover, the time gap between GAS infection and the occurrence of ARF is variable, and therefore many patients do not recall having had pharyngitis. Therefore ARF is usually underdiagnosed in developing countries.^26^ Additionally, many patients are not correctly treated, secondary prophylaxis is not instituted and progression to RHD occurs, explaining the high incidence of newly diagnosed RHD in adults.^27^

Subclinical disease is commonly found in Africa when echocardiographic screening is used.9-11 The advent of portable battery-powered ultrasound machines has allowed access to the communities and recognition of the need for an update of the WHO criteria for echocardiographic diagnosis of subclinical RHD. It has been suggested that in endemic areas the diagnosis can be based on the presence of pathological valve regurgitation without considering the morphological features of the valves.^28^

African scientists were also part of the World Heart Federation panel of experts that created a set of screening criteria using morphological and Doppler features, aimed at standardising the diagnosis across different areas of the world.^29^ Therefore, although echocardiographic diagnosis of RHD is not yet readily available in some parts of Africa, its use has allowed better characterisation of cardiac abnormalities, definition of the natural history of the disease and assessment of the current practice in managing these patients on the continent. The Global Registry of RHD^30^ confirmed the extremely virulent forms of chronic RHVD in Africa.

RHD in Africa is encountered in young people, showing a much faster and malignant progression of cardiac involvement than that seen in Europe or North America.^31^ Severe disease and rapid progression to complications such as mitral stenosis, heart failure and atrial fibrillation occur at younger ages.^30^-^32^ It is believed that this pattern results from environmental factors such as higher occurrence of skin and pharyngeal streptococcal infections in these settings, recurrent GAS infections early in life, inadequate treatment of GAS infections, and inappropriate secondary prophylaxis after the first episode of RF, but the role of host specificity in determining the malignant course of the disease in Africa cannot be excluded.

Benzathine penicillin G (BPG), the gold standard for secondary prophylaxis of RF/RHD, is usually administered every three or four weeks. Occurrence of ARF in patients on adequate secondary prophylaxis with BPG has been attributed to the low quality of the product, inadequate storage, inappropriate technique for injection, and incorrect dosage for the patient’s weight.^33^

Knowing that HLA-DRA variants were found to predict penicillin allergy in genome-wide fine-mapping genotyping, one may speculate on the need to explore whether genetic polymorphisms determine differences in pharmacokinetics and/or pharmacodynamics of penicillin in African individuals. This is of particular relevance considering that the correct management of GAS pharyngitis and secondary prophylaxis of RF with penicillin prevent the occurrence of RHD. Currently, there are no data to support a higher occurrence of penicillin allergy in Africa than is seen in other parts of the globe. However, of importance for the implementation of control programmes in Africa, it has been suggested that analysis of gene variants of HLA-DRA and the HLA-DRA|HLA-DRB5 inter-region, which may be significant predictors of allergy to penicillin, should occur in African populations.^34^

## The role of genetic studies

ARF and RHD are caused by a combination of immune, environmental and genetic factors. While the role of GAS and social conditions that determine progression to RHD is well understood,^35^,^36^ there is a major gap in knowledge of the mechanisms of host susceptibility to the disease.^28^ Familial aggregation, similarity of disease patterns between siblings, concordance of disease in identical twins, and HLA correlation studies are evidence for a genetic influence on RF susceptibility.^37^ A systematic review and meta-analysis of 435 twin pairs from six independent studies concluded that ARF has high heritability, estimated at 60% across all the studies; the pooled proband-wise concordance risk for ARF was 44% in monozygotic twins and 12% in dizygotic twins.^38^

Only 0.4 to 3.0% of patients with untreated GAS pharyngitis develop ARF, but higher attack rates occur when a stronger host immune response occurs, approaching 50% in patients with a prior episode of ARF. In patients with the first episode of ARF, the rate of progression to RHD will differ, probably being related not only to environmental factors such as the high recurrence of GAS and different virulence of the circulating GAS, but also to a particular immune response geared by genetic susceptibility.^37^ Similarly, the genetic background directing the immune response towards a predominantly Th1 or Th2 pattern may contribute to explain variations in RF clinical phenotype by modulating the intense and sustained inflammation that is needed to cause sequelae such as RHD.^17^,^37^

Inherited susceptibility to ARF was initially studied around the major histocompatibility class II human leucocyte antigens (HLA). Several genes associated with RHD have been described, most of them involved with immune responses.^39^ Given the current state of the literature, it is hard to make generalisations about a single ‘rheumatic’ HLA allele, and there are likely multiple HLA alleles that, in combination, increase an individual’s susceptibility to ARF and RHD.^2^

HLA-D8/17 and HLA-DR7 types are the most represented in the literature, but many other HLA alleles have been identified in single studies of patients with ARF and RHD (Table 1), a variability that could be caused by genetic differences in the populations studied or differences in local streptococcal strains. A study in Uganda comparing the frequency of HLA class II DR alleles between RHD cases and healthy controls found HLA-DR1 to be more common in normal controls while HLA-DR11 was more common among RHD cases.^1^ Candidate HLA gene studies that have been performed to date had small sample sizes and found inconsistent and conflicting results.^36^,^40^ High-resolution HLA analysis and genome-wide association studies have therefore been recommended.

**Table 1:** RHD genetic susceptibility; HLA class II alleles found in studies in patients from different regions of the globe (adapted from Guillerme *et al.*^39^ and updated).

*Continent*	*Country, reference*	*HLA class II alleles*
Africa	South Africa	DR1, DR6
	Uganda	DR1, DR11
	Egypt	DRB1*0701, DQA1*0201, DRB1*13, QA1*0501/0301
Americas	United States of America	DR2 (Africans); DR4, DR6, DR9 (Caucasians)
	Mexico	DRB1*1602, DQB1*0301, DQA1*0501
	Martinique	DR1
	South Brazil	DR7, DR53
	India	DR3
	Kashmir	DR4
	Japan	DQB1*05031, DQA1*0104
	South China	DQA1*0101
	Saudi Arabia	DR4
Americas	Turkey	DR3, DR7, DR11
Europe	Latvia	DRB1*0701, DQB1*0302, DQB1*0401-2

Single-nucleotide polymorphisms in a number of genes were found in patients with RHD compared to controls, namely protein tyrosine phosphatase non-receptor 22 (PTPN22),^41^ signal transducers and activators of transcription (STAT),^42^ angiotensin converting enzyme (ACE I/D),^43^ TNF-α,^44^,^45^ transforming growth factor (TGF-β1),^46^,^47^ and TLR5^48^ (Table 2). Studies in North Indians with RHD suggest that the (PTPN22) haplotype, which encodes an important negative regulator of T-cell activation, modulates the risk of developing RHD.^41^ In a Turkish population, however, it was demonstrated that the PTPN22 R620W polymorphism was not associated with RHD,^49^ showing that genetic differences exist among populations from different regions of the world, therefore making it relevant to implement similar studies in Africa.

**Table 2:** Genes found in ARF/RHD studies. The country and number of participants are indicated.

*Author, year*	*Country*	*Sample size*	*Genes*
Gupta et al. 2014^41^-^43^	India	400 RHD patients	PTPN22 polymorphisms
		300 controls	JAK/STAT polymorphisms
		300 RHD patients	ACE I/D polymorphisms
		200 controls	
Aksoy et al. 2011^49^	Turkey	120 RHD	PTPN22 R620W gene polymorphism
		160 controls	
Mahomed et al. 2010^44^	Saudi Arabia	80 RHD patients	TNF-α polymorphisms
		50 controls	
Kamal et al. 2010^47^	Egypt	73 RHD patients	TGF-β1 polymorphisms
		55 controls	

Overall, the current knowledge of genetic susceptibility for RHD comes from small studies (Table 2). Moreover, because subclinical disease is frequent in Africa and RHD is diagnosed in the late stages, it is related to high morbidity rates, premature mortality and excessive social and economic costs. The finding of genetic biomarkers could direct the scarce resources available on the continent to those persons at higher risk, thus reducing the workload of health professionals, avoiding the high burden related to this condition, and improving outcomes.

The Global Registry of RHD30 represents a platform for such genetic studies on RHD in Africa. These studies will improve our understanding of genomic and epigenetic drivers of heterogeneity in the response of different individuals to GAS infections, and explore the determinants and drivers of the variability in natural history, clinical phenotype, prognosis, and the role of genetic differences in determining allergy and drug resistance to penicillin in sub-Saharan Africa. The discovery of genetic susceptibility loci through whole-genome scanning may be clinically useful by introducing genetic risk-prediction tools for ARF and RHD.

## Key messages

• ARF, the precursor of RHD, is usually underdiagnosed
• RHD is highly prevalent in Africa, where it affects much younger people
• ARF and RHD are caused by a combination of immune, environmental and genetic factors
• The magnitude of the genetic effect remains unclear but high heritability has been shown
• Africa has the most virulent and rapidly progressive forms of ARF and RHD
• Genetic studies may help to explore determinants of variability in the natural history and phenotype.

## Conclusion

Research to determine the role of genetic factors in determining susceptibility to ARF and RHD in African populations is needed. These genetic studies in the African context may contribute to a greater understanding of the genomic and epigenetic drivers of heterogeneity in individual responses to GAS infections and progression to RHD. Discovery of genetic susceptibility loci through whole-genome scanning may provide a clinically useful genetic risk-prediction tool that will potentially allow echocardiographic screening and secondary prophylaxis to be directed to those at higher risk, thus reducing the burden of the disease on the health system, the work health force and the communities of this resource-strained continent.
